# Mathematical calculation of the difference in shortening length after two types of proximal femoral varus and an investigation of their applicable conditions: an own-pair design

**DOI:** 10.1186/s13018-022-03462-1

**Published:** 2022-12-23

**Authors:** Jian Sun, Yong Cui, Jing Qu, Feng Lian

**Affiliations:** Department of Orthopaedics, The Fourth Hospital of Harbin Medical University, 37 Yiyuan Street, Nangang District, Harbin City, Heilongjiang Province China

**Keywords:** Developmental dysplasia of the hip, Varus osteotomy, Femoral shortening, Bilateral lower extremity inequality, Trigonometry

## Abstract

**Background:**

The shortening length of the lower extremity after the proximal femoral osteotomy is an important issue to be considered in preoperative planning of developmental dysplasia of the hip (DDH) in children. There is still a lack of research on shortening the length of the lower extremities in different proximal femoral osteotomy varus styles. We aimed to verify the relationship between the shortening length after “point-to-face” and “face-to-face” varus osteotomy and proposed a formula for calculating the difference in shortening length and verified its feasibility.

**Methods:**

Fifty-five children with unilateral DDH were enrolled. The preoperative hip CT data were imported into mimics 21, 3-Matic 10 (Materialise, Leuven, Belgium) for femoral reconstruction and simulated osteotomy, and the difference (*t*) was calculated by directly measuring the length of the proximal femur after osteotomy. d* sin*θ* was measured in a three-dimensional environment to calculate the difference in femoral shortening length between the two osteotomy methods (*t*').

**Results:**

The results of the direct measurement method and the formula measurement method are shown in the table; the differences in the results of the femoral shortening length difference were not statistically significant (*P* > 0.05). The limits of agreement (95%) of the difference values using Bland–Altman analysis were between − 0.50 and 0.46 mm, with a mean of − 0.02 mm, indicating a high agreement between the two methods. *r* = 0.99 (*P* < 0.05) for the Pearson correlation analysis between the direct measurement method and the calculated method showed that the two methods were significantly correlated.

**Conclusions:**

The derived formula can accurately calculate the difference in the shortening length of the proximal femur after “point-to-face” and “face-to-face” varus osteotomy in children with DDH, which is suitable for clinical application.

## Introduction

DDH is one of the common hip disorders in pediatric orthopedics, and the choice of treatment depends on the age of the child and the severity of the disease [1, 2]. Infants up to 6 months of age who are confirmed to have hip instability or dislocation are generally treated with a brace initially, such as a Pavlik harness or abduction orthosis. Patients aged 6 to 18 months with dislocation can be treated with closed reduction and the application of a hip spica cast. Generally, patients > 12 to 18 months of age or those who fail to achieve a concentric hip reduction with closed methods are considered candidates for open surgical hip reduction. Osteotomies, such as femoral shortening osteotomy and pelvic osteotomy, are considered for hip dislocation in older patients to decrease tension on the hip reduction and those with a residual shallow dysplastic acetabulum, respectively [3].

**Fig.1:**
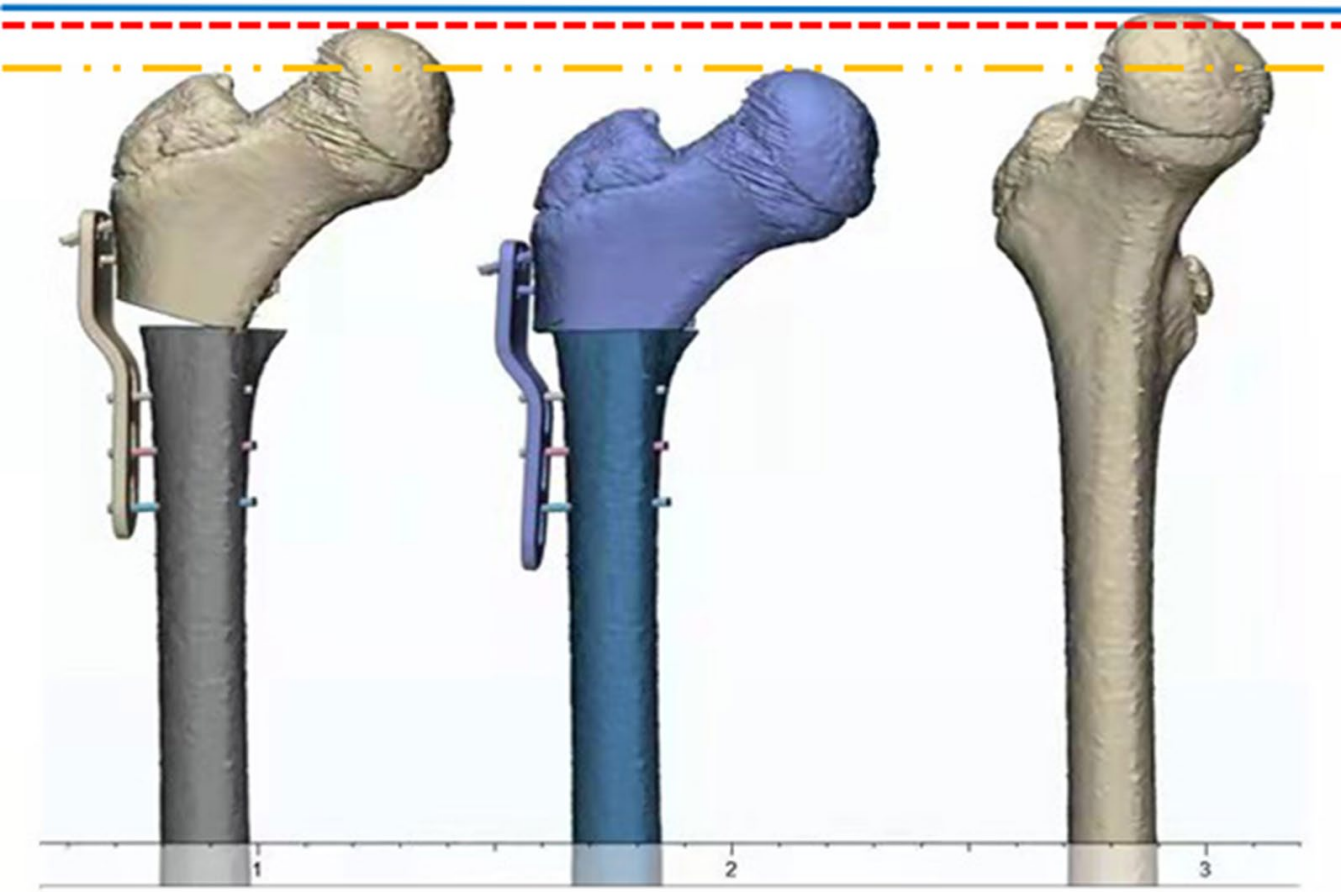
Comparison of femoral length before and after osteotomy. Between the blue line and the red line is the length of femoral shortening after “point-to-face” and osteotomy. Between the blue line and the yellow line is the length of femoral shortening after “face-to-face” osteotomy

Children with DDH often present with acetabular dysplasia and femoral anatomical abnormalities, including hip valgus and increased femoral anteversion [4], and the concentric relationship of the head and socket is restored through varus osteotomy to increase the acetabular accommodation of the femoral head. Good accommodation allows the femoral head to obtain optimal bioplasty and increase hip mobility and can stimulate the normal development of the acetabulum; at the same time, varus can relax the iliopsoas muscle, hip abductor muscle, adductor muscle group, and rectus femoris and reduce the pressure on the head socket. The closing wedge technique is commonly thought to offer the greatest mechanical stability [5]. Femoral length change after osteotomy in children with DDH is an indicator that needs to be clarified by the operator, and there is a lack of comparative studies on femoral length after different osteotomy approaches. In this study, the horizontal osteotomy and wedge osteotomy were called “point-to-face” and “face-to-face” osteotomy, respectively, and according to the way the distal and proximal osteotomies were combined after osteotomy. In our preliminary study of the two types of varus, we found that the shortening of the proximal femur was always greater in the “face-to-face” osteotomy than in the “point-to-face” osteotomy for the same angle of varus (Fig. 1). The varus osteotomy often leads to medical inequality of both lower extremities, causing complications such as claudication, compensatory scoliosis, hip abduction dysfunction, and low back pain [5].

We studied the operation procedure and postoperative proximal femur morphology of two types of varus osteotomy and derived the trigonometric equation for the difference in femoral shortening length between the two osteotomy methods and verified its feasibility by simulating the osteotomy on the side of 55 children with DDH.

## Patients and methods

### Clinical data

The clinical data of 55 children with unilateral DDH (55 hips) admitted to our hospital from January 2010 to June 2021 were collected retrospectively according to the inclusion and exclusion criteria, including 13 males and 42 females, aged 2–10 years (mean 6.2 years); 22 cases on the left side and 33 cases on the right side. There were 15 cases of Crowe type I, 19 cases of type II, 13 cases of type III, and 8 cases of type IV.

This study was approved by the ethics committee of the Fourth Affiliated Hospital of Harbin Medical University (approval no.2022-SCILLSC-05) and met the requirements of the Declaration of Helsinki. The legal representative of each patient signed the informed consent.

All patients should meet the following criteria:

Inclusion criteria were: (1) children with unilateral DDH; (2) age 2–10 years; (3) no history of hip infection; (4) no history of femoral shortening osteotomy or epiphyseal block surgery; (5) no obvious hip and knee flexion deformity; and (6) complete bilateral hip CT information before surgery. Exclusion criteria were: (1) lack of preoperative hip CT information; (2) flat hip deformity (Perthes disease); (3) post-traumatic hip dislocation; (4) hip dislocation secondary to cerebral palsy and other disorders; (5) children with bilateral DDH; and (6) other causes of unilateral limb dysplasia and dislocation.

### Methods

All patients underwent their own-paired design, and their proximal femurs were subjected to computer-simulated “point-to-face” and “face-to-face” varus osteotomies, and the difference in femoral shortening length was obtained using direct and formulaic measurements. The results of the direct measurement method were recorded as *t*, and the results of the formula method were recorded as *t*'.

**Fig. 2:**
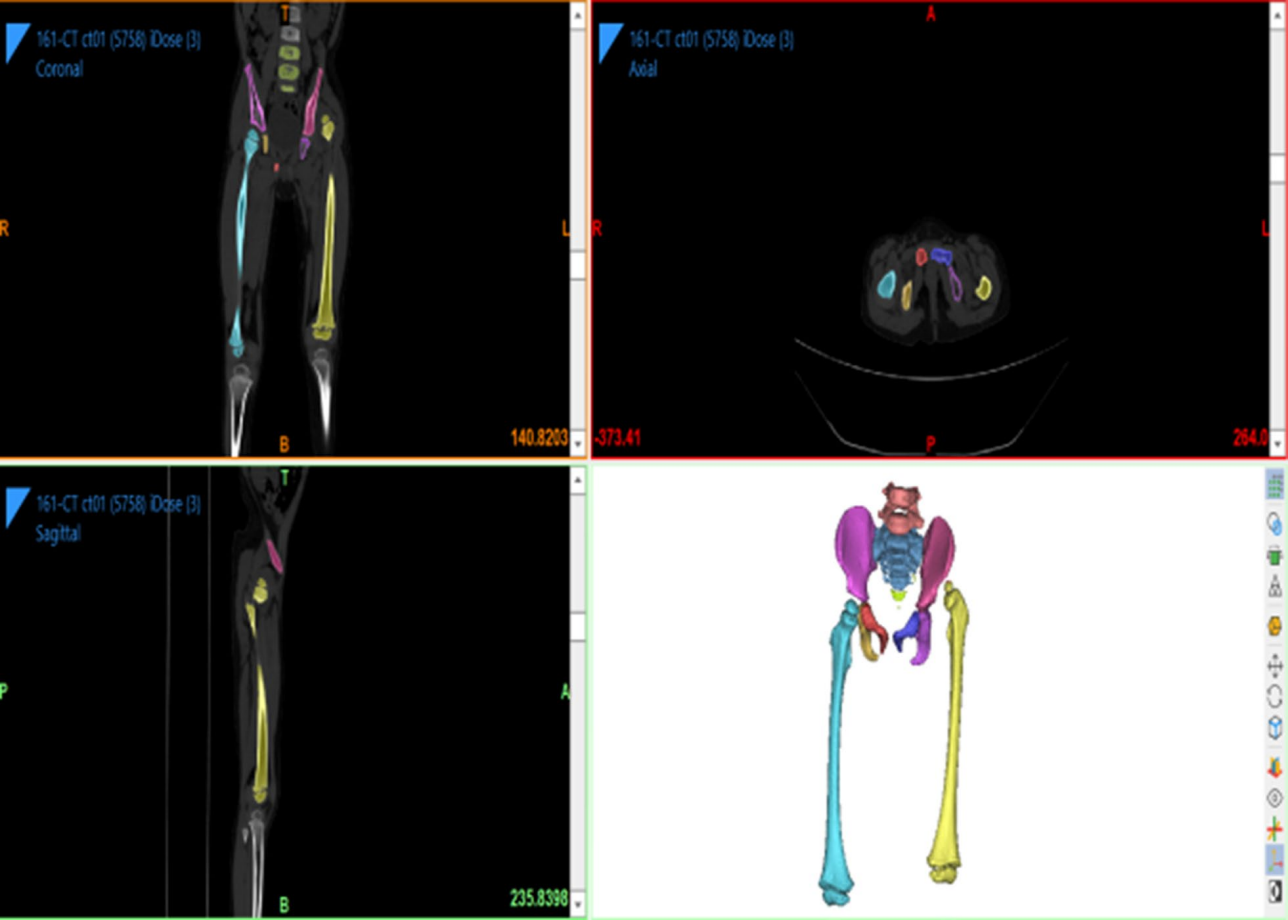
The patient’s CT data are imported into the software, and a mask is created. The original mask is segmented using the region growth command, so that each part of the bone is extracted separately

**Fig. 3:**
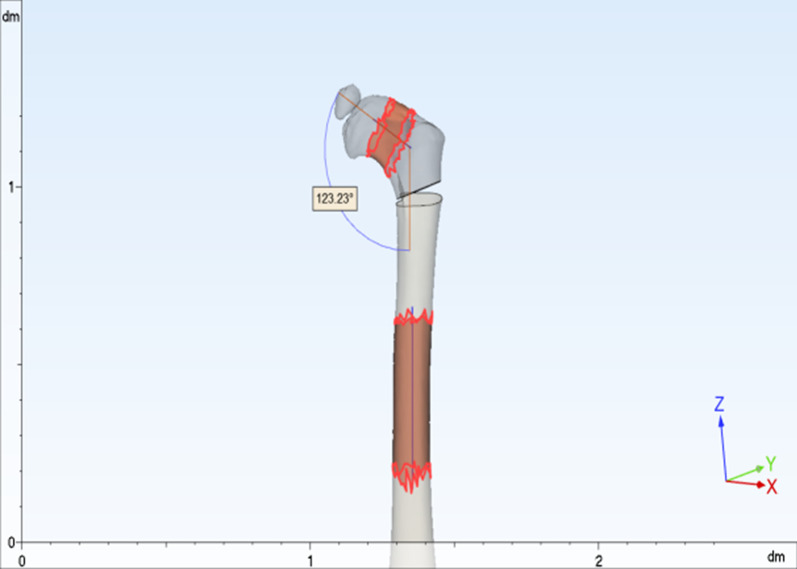
Selection and marking of the femoral neck and femoral stem with uniform diameter, fitting the femoral neck and femoral stem axes, and measuring the neck–stem angle in 3D space, i.e., the angle between the fitted femoral neck axis and the femoral stem axis

**Fig. 4:**
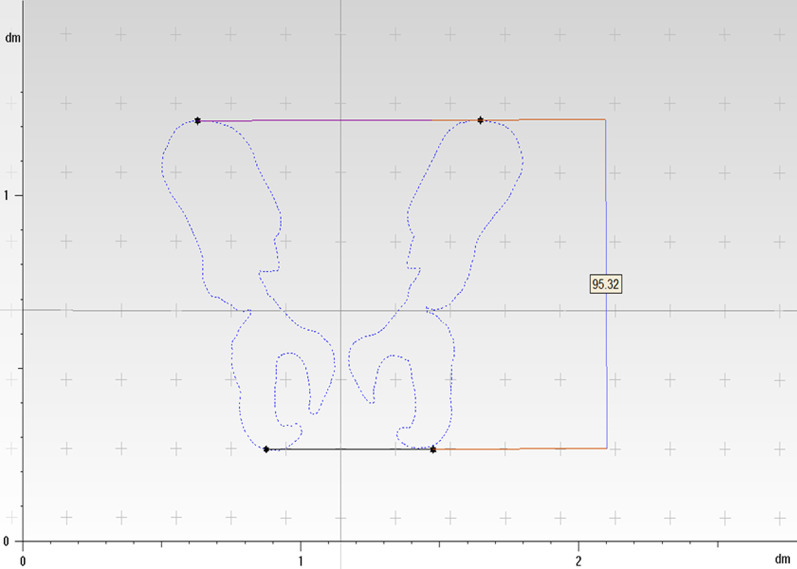
The distance between the line connecting the highest point of the iliac crest bilaterally and the line connecting the lowest point of the ischial tuberosity bilaterally is the pelvic height, by taking the standard coronal plane projection of the patient’s pelvis

**Fig. 5:**
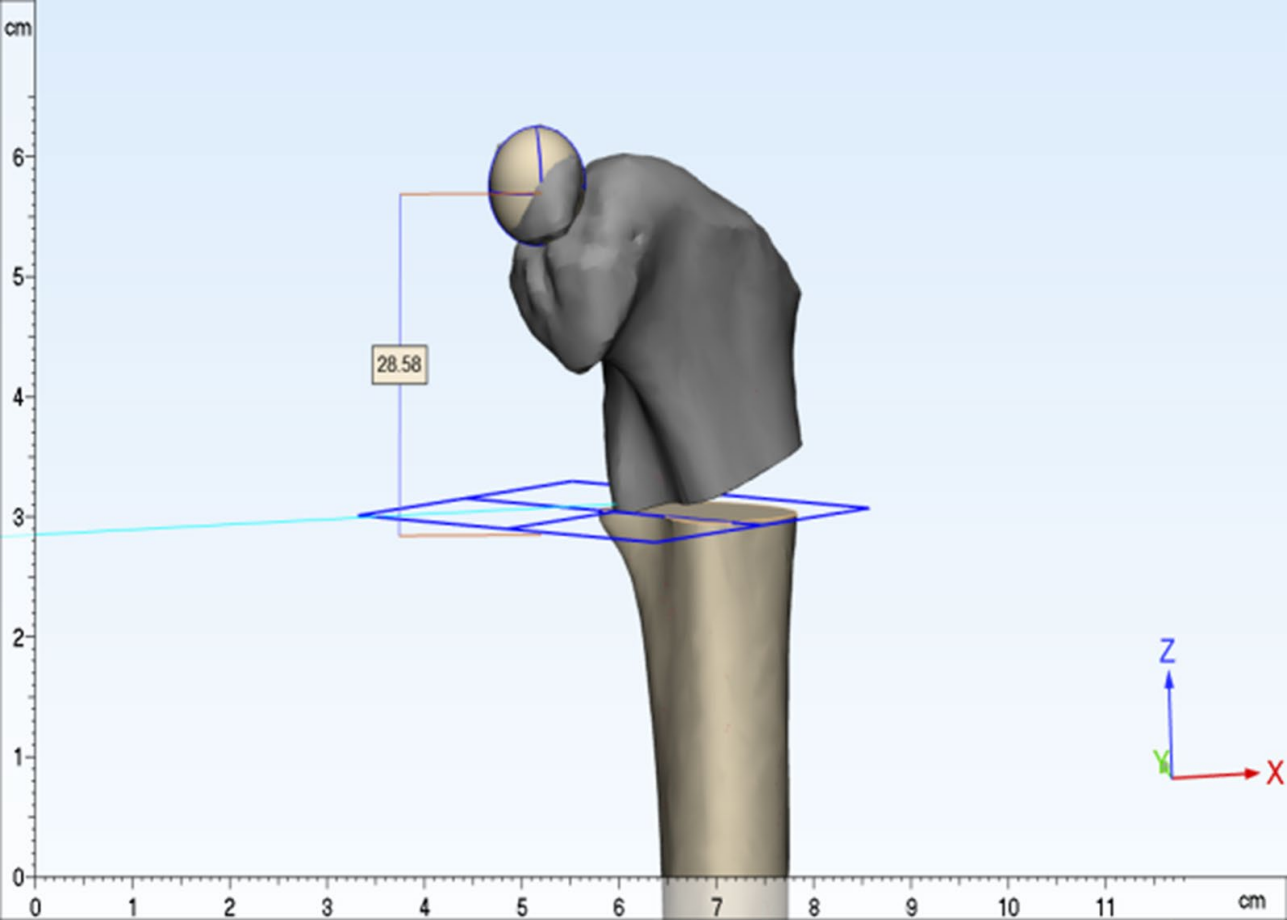
The femoral head is spherically fitted, and the center of the sphere is used as the center of the femoral head. The plane in which the center of the lesser trochanter is located is used as the osteotomy plane, and the proximal femoral varus is performed according to preoperative planning. The distance from the center of the femoral head to the osteotomy plane is the distance of the proximal femur after osteotomy

**Fig. 6:**
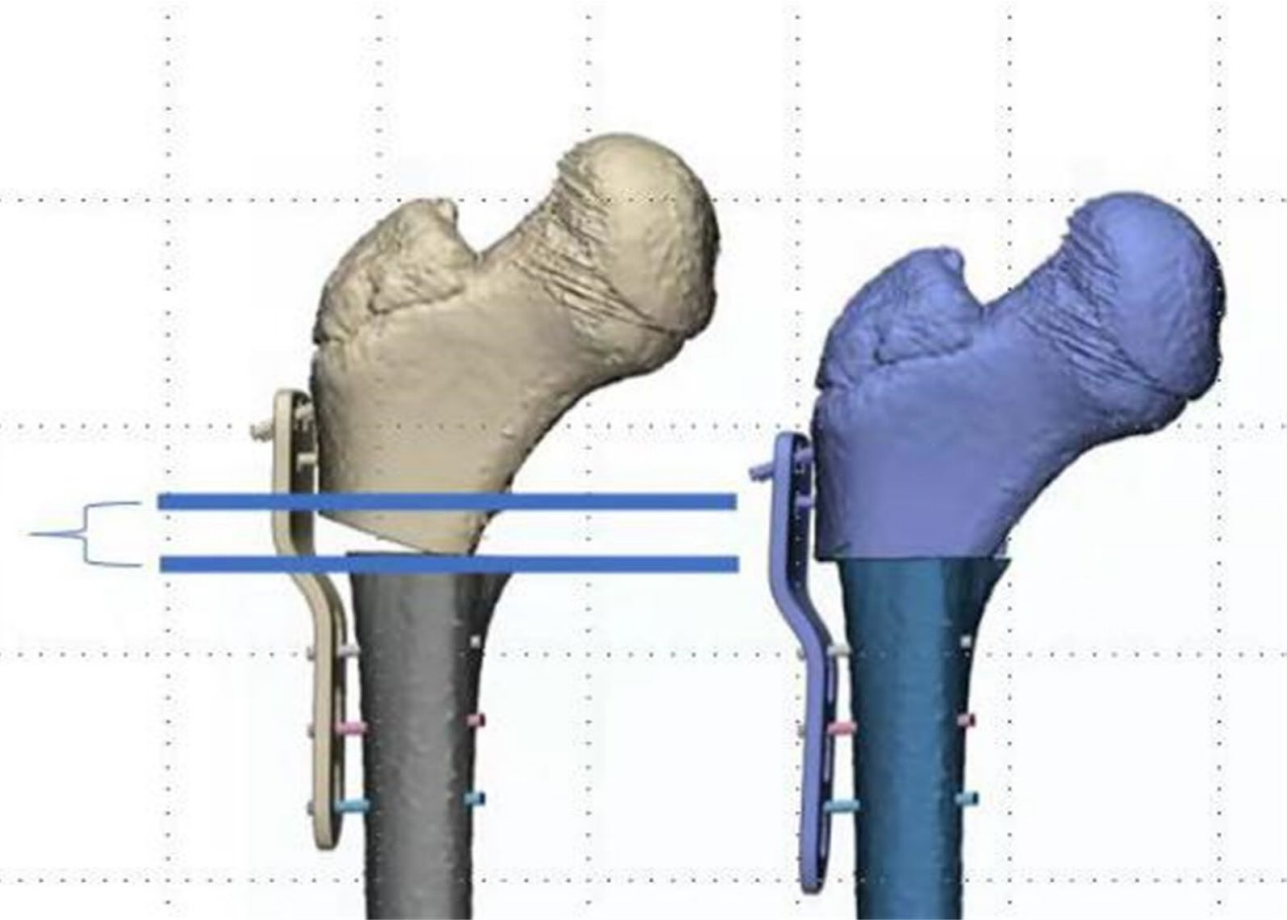
The figure shows the proximal femur after “point-to-face” and “face-to-face” varus osteotomy. The distance between the two blue lines is the length difference between the two types of osteotomies

**Fig. 7:**
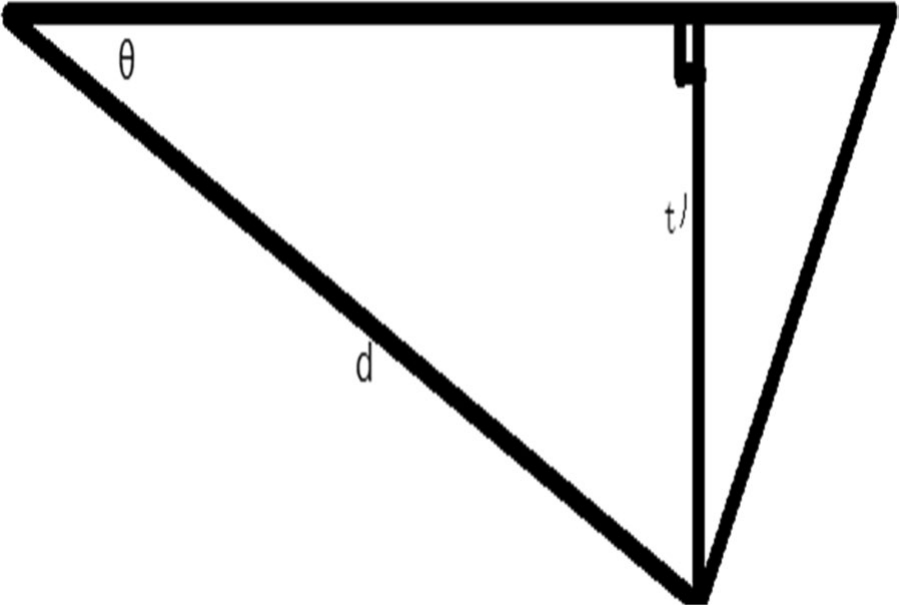
The figure is a line drawing of the difference between the “point-to-face” and “face-to-face” osteotomy in Fig. 6. *θ* is the proximal femoral varus angle. *d* is the width of the femur at the osteotomy surface, and t' is the theoretical shortening length between the two types of varus

1. Direct measurement of* t*: All patients were scanned by Philips Brilliance 64-slice spiral CT (scanning conditions: slice thickness 1.0 mm, slice interval 0.5 mm, tube voltage 120 kv, tube current 90–150 mA) at the Fourth Affiliated Hospital of Harbin Medical University. The patient is in a neutral supine position with the patella facing the ceiling. Scans were taken from the ilium to the medial tibia. All standard CT slices were preserved in Digital Imaging and Medical Communications (DICOM) format and imported into Mimics 21.0 software (Materialise, Leuven, Belgium) for 3D reconstruction.
  The CT data of the child in DICOM format were imported into mimics 21.0 software, the bone threshold was extracted, and the original mask was created; the left femur, ilium, situs and pubis, the right femur, ilium, situs and pubis, and the sacrococcygeal mask were extracted by using the region growth command, and the mask editing, filling, and smoothing operations were performed, respectively (Fig. 2); the 3D entities were calculated and imported into 3-matic11. import into 3-matic11; use the rectangle marker command to coarse and thin the femoral neck and femoral body and fit the neck axis and stem axis, and measure the angle between the neck axis and stem axis in 3D space, that is, the neck–stem angle (Fig. 3); create a sketch plane parallel to the coronal position of the pelvis, use the file import command in the sketch to generate a 2D view of the pelvis projected on this plane, and create points at the highest point of the iliac crest on both sides and the lowest point of the sciatic tuberosity. The distance between the two lines measured is the pelvic height of the child m (Fig. 4); according to the varus angle in the child’s surgical record, the sketch and the osteotomy line with the corresponding angle are created at the femoral osteotomy line (at the level of the midpoint of the lesser trochanter), and the osteotomy is performed according to the osteotomy line in the sketch (“face-to-face” style) or at the femoral osteotomy line (at the level of the midpoint of the lesser trochanter). The proximal femoral varus follows the straight line in the sketch (“point-to-face”); the analysis of sphere function in the design menu is used to fit the ossified portion of the femoral head to a sphere, and the center of the fitted sphere is used as the center point of the femoral head. The distance between the center point of the femoral head and the plane of the osteotomy line was measured using the distance measurement function in the measurement menu for the “point-to-face” (*t*1) (Fig. 5) and “face-to-face” (*t*2) varus osteotomies, and the postoperative femoral length difference *t* (*t* = *t*1−*t*2) was calculated.
2. Formula to calculate *t*': According to the “point-to-face” and “face-to-face” surgical procedures and postoperative proximal femur morphology (Fig. 6), the femoral width at the level of the osteotomy line is defined as d, and the proximal femoral varus angle is *θ*. The simulated osteotomy model and simplified diagram can be derived from the postoperative femur length difference *t*' = d*sin*θ *(Fig. 7). *d* to measurement was performed in the anterior view of the femoral model, and the varus angle θ could be obtained according to the surgical records of the child, and the t' value could be calculated by bringing it into the above formula.
3. Statistical methods
  SPSS26.0 statistical software (IBM Corp, Armonk, NY, USA) was used to process the data, and the measurement data were expressed as Mean ± SD. The coefficient of variation (CV) to indicate the dispersion of the two sets of results is obtained by dividing the sample standard deviation by the mean value. Paired t test was used to compare the results obtained by the direct measurement method and formula method, and *P* < 0.05 by a two-sided test was considered as a statistically significant difference. Pearson correlation analysis was performed to correlate the direct measurement method with the computational method. Bland–Altman consistency analysis was performed for both methods using MedCalcV15. 2. Data analysis software (MedCalc Statistical Software, Belgium). Since the t values obtained by the direct method depended on the measurement of the distance n1 and n2 from the center of the ball to the plane of the osteotomy line after the “point-to-face” and “face-to-face” varus osteotomy, the t' values for each child in the formula method depended on the measured femoral width d at the level of the osteotomy line. The effect of human factors on the measurement data was analyzed: One measurer measured the above indexes twice at an interval of 1 week; two measurers measured the above indexes once each. Reproducibility and reliability were expressed as excellent, good, and poor using the ICC (intra-class correlation coefficient), where > 0. 75 is excellent, 0. 4–0. 75 is good, and < 0.4 is poor.

## Results

**Fig. 8:**
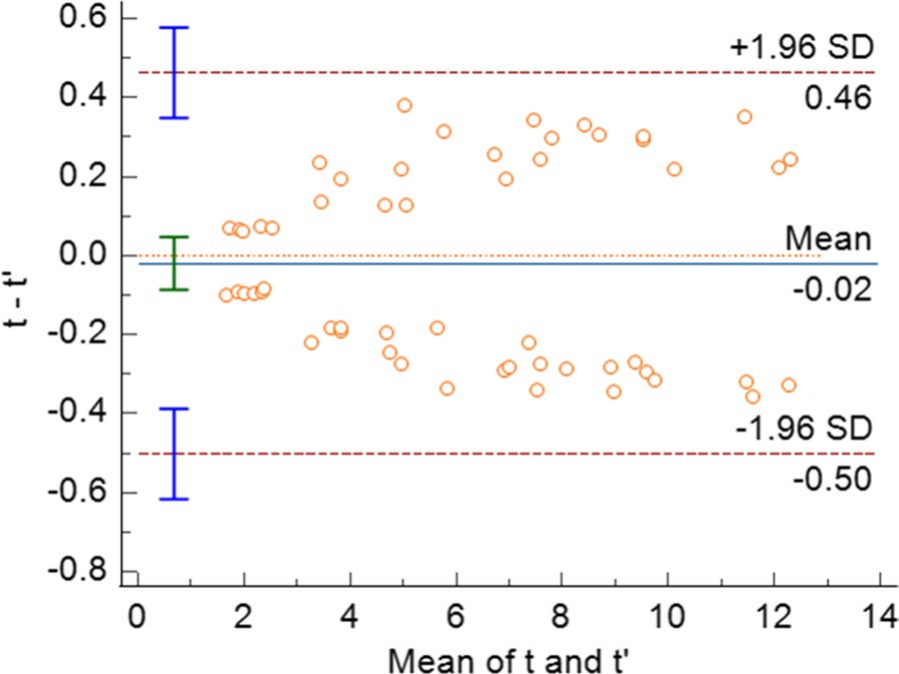
Results of the Bland–Altman consistency analysis using the values obtained by the direct measurement method and the formula method

The shortening length mean ± SD of the proximal femur in 55 children was 6.3 ± 3.2 mm by direct measurement and formula method, and the CV was 50.79%. The statistical results at different valgus angles are shown in Table 1. The difference in femoral shortening length between the two types of osteotomy was not statistically significant (*P* > 0.05). Using Bland–Altman analysis of the values obtained by the direct measurement method and the formula method, the limits of agreement (95%) for the differences between the two methods were between − 0.50 mm and 0.46 mm, with a mean of − 0.02 mm, indicating a high agreement between the two methods (Fig. 8). Pearson correlation analysis of the direct correlation coefficient *r* = 0.99 (*P* < 0.05) between the measurement method and the formula method showed that the two methods were significantly correlated. The repeatability and reliability of the measurements of the same measurer (ICC(*t*) = 0.995, ICC(*t*') = 0.997) and two measurers (ICC(*t*) = 0.996, ICC(*t*') = 0.995) were excellent (Table 2).

**Table 1:** Two groups of measurement results under different varus angles

Angle	*T *(*n* = 55)		*t*' (*n* = 55)		*t*−*t*' & 95%CI	*P* value
	Mean ± SD (mm)	CV(%)	Mean ± SD (mm)	CV(%)		
5°(*n* = 11)	2.1 ± 0.3	14.29	2.1 ± 0.3	14.29	− 0.02 (− 0.3–0.2)	0.9
10°(*n* = 11)	4.1 ± 0.7	17.07	4.0 ± 0.6	15.00	0.01 (− 0.6–0.6)	0.98
15°(*n* = 11)	6.2 ± 1.1	17.74	6.3 ± 1.1	17.46	− 0.07 (− 1.0–0.9)	0.9
20°(*n* = 11)	8.6 ± 1.3	15.12	8.5 ± 1.3	15.29	0.02 (− 1.1–1.1)	0.97
25°(*n* = 11)	10.4 ± 1.7	16.35	10.4 ± 1.7	16.35	− 0.04 (− 1.6–1.5)	0.96

CI = confidence interval; CV = coefficient of variation

**Table 2:** Consistency test of* t* and* t*' results under human factors

measurer	The ICC of *t*	The ICC of *t*'	*P* value
The same measurer	0.995	0.997	0.00
Two measurers	0.996	0.995	0.00
*P* value	0.00	0.00	–

ICC = Intra-class correlation efficient

## Discussion

DDH treatment is largely related to the age of the patient. In the child with hip dysplasia or frank dislocation, increasing age is associated with impediments to reduction, as well as increased soft tissue contracture or residual bony deformity [6]. Proximal femoral varus osteotomy can delay or even avoid the need for hip replacement in patients. Ansari et al. concluded that proximal femoral varus osteotomy significantly increased the Harris hip score in patients with DDH who failed conservative treatment [7]. Although it is widely accepted, current studies have found that excessive femoral shortening can cause serious problems and affect long-term surgical outcomes; for example, varus osteotomy of the proximal femur may lead to relative shortening of the femur; excessive shortening can lead to claudication, as well as hip abduction dysfunction, compensatory scoliosis, low back pain, and other complications due to the center of rotation of the hip being below the level of the greater trochanter, which affects the long-term surgical outcome.

Lower limb overgrowth after DDH open osteotomized surgery has been identified. Chan Yoon et al. suggested that this overgrowth phenomenon might be the same pathogenic mechanism as overgrowth after femoral stem fracture [8]. In skeletally immature children, it is generally considered acceptable to have bilateral lower extremity inequalities within 1 cm. Similarly, Segaren et al. indicated that, in their clinical experience, overgrowth of approximately 1 cm can occur in the affected limbs of children younger than 10 years of age [5]. In a multicenter study on the treatment of femoral fractures in children, an evaluation index was proposed for the phenomenon of femoral overgrowth, and it was concluded that the prognosis was excellent for patients with a bilateral lower limb length difference of 1 cm or less, satisfactory for those with 1 to 2 cm, and poor for those with more than 2 cm [9]. Therefore, it is necessary to study the length of the shortening and the procedure of osteotomy for proximal femoral varus. Precise preoperative planning and surgical operation can reduce the occurrence of postoperative complications such as bilateral lower extremity inequality and improve the prognosis of the child.

The morphology of the proximal femur is complex, and the clinical measurement of the nuchal stem angle and other related parameters is often performed with the help of X-ray, which is two-dimensional imaging and simple to operate, but due to the existence of the anterior inclination angle of the proximal femur, the measurement of the nuchal stem angle in the two-dimensional plane inevitably has some errors. Anastopoulos et al. [10] found that the coronal projection of the femoral neck–stem angle was on average 5°larger than its 3D value after 3D modeling of 22 cadavers. Bonneau et al. [11] measured the difference between the femoral neck–stem angle and X-ray measurements in 91 European subjects using a 3D model with a mean value of 4.5°(2.4°-6.0°). Computer-aided measurement has obvious advantages, especially for length measurement, which improves the accuracy of positioning of measurement marker points and thus the accuracy of measurement. In this study, a three-dimensional model of the femur was established based on CT data, and the measurements of the neck–stem angle and the distance between the center point of the femoral head and the osteotomy surface after osteotomy varus were all three-dimensional measurements, which to a certain extent avoided the influence of measuring the anteversion angle of the femoral neck in pelvic plain films on the results. The correlation analysis of the measurement results also showed that the results were significantly reproducible and reliable, making up for the shortcomings of the traditional measurement methods.

**Fig. 9:**
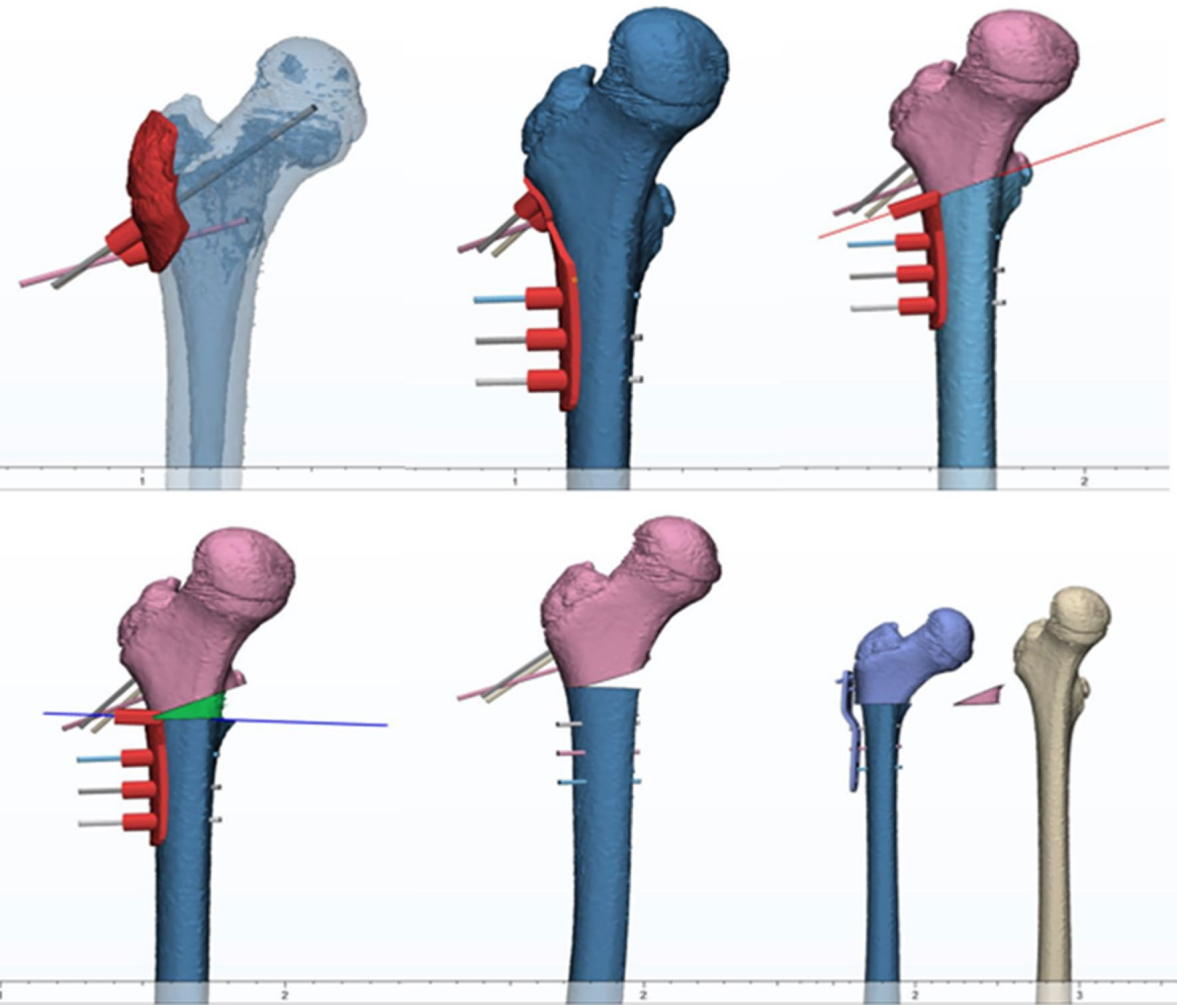
Diagram of the operation of the “split” guide for “face-to-face”

Sub-rotor is currently the more commonly used location for proximal femur varus osteotomy, and the corresponding rotational osteotomy can be performed at the same time according to the need. According to the different osteotomy methods, there are cuneiform osteotomy, horizontal osteotomy, oblique osteotomy, etc. The horizontal osteotomy is simple, easy to perform, and can be performed at the same time. Among them, horizontal osteotomy has the advantages of simple operation and short operation time, which is widely used in clinical practice, but there is a risk of poor rotational stability and non-healing of the osteotomy surface. Wedge osteotomy has obvious advantages in terms of mechanical stability and bone healing, but due to the difficulty of operation, steep learning curve, the size of the correction angle mainly depends on the operator’s experience, so it is easy to cause the loss of correction angle, affecting the treatment effect, and the clinical application is relatively small. The success and precision of proximal femoral varus rotation and shortening osteotomy is mainly based on the experience of orthopedic surgeons [12]. We successfully designed and printed a “split” guide for “face-to-face” assisted osteotomy using computer-aided design and 3D printing technology and applied for a patent for the invention. Our invention consists of 4 auxiliary osteotomy guides and several Kirschner wire centering device of the same size. The 4 auxiliary osteotomy guides consist of one positioning guide, one connecting guide, one oblique osteotomy guide, and one transverse osteotomy guide. The procedure is summarized as follows: Three Kirschner wires are placed with the aid of the proximal femoral positioning guide; the connecting guide is installed through the proximal  three Kirschner wires; the oblique osteotomy guide is placed through the distal  three Kirschner wires and the bone is osteotomized; the transverse osteotomy guide is placed through the distal  three Kirschner wires and the bone is osteotomized; and the osteotomized bone is removed and the plate is fixed (Fig. 9). In vitro 3D models of osteotomy and clinical applications have shown that the use of this “split” guide significantly reduces the difficulty of osteotomy and allows for significant accuracy and ease of operation.

There are few studies on the prognosis and complications associated with the use of different osteotomies on the femoral side. Closed wedge osteotomies are generally considered to provide the greatest mechanical stability and reduce the incidence of internal fixation failure and postoperative nonunion. For “point-to-point” and “face-to-face” osteotomy, theoretically, the latter has a larger contact area to ensure bone healing, while the former tends to cause stress concentration at the contact area. However, further statistical analysis of the clinical data is needed to determine whether there is any significant difference in the actual prognosis and complications. At present, the choice of femoral osteotomy method mostly depends on the clinical experience of the surgeon, and no consensus has been formed. However, the shortening length of the affected limb was not the same with the same angle of varus in different procedures. We found that the shortening length of “face-to-face” was greater than that of “point-to-face” with the same angle of varus, and a simple calculation method was also given in this study. Based on this, we discussed the conditions of the two varus methods to guide the clinical selection of the appropriate one.

The pelvic height of children with DDH aged 2–10 years measured by us was (96.76 ± 9.43) mm. Crowe typing is the current common clinical method of DDH typing; based on it and our measured pelvic height (averaged) we can roughly calculate the distance of femoral head displacement in children with Crowe I, II, III, and IV which can be roughly calculated as < 9.68 mm, 9.68–14.51 mm, 14.51–19.35 mm, and > 19.35 mm, respectively [13]. Liu XM et al. studied the relationship between the “point-to-face” varus angle and the shortening length of the proximal femur in children aged 5–10 years and found that the shortening length was about 2 mm for varus angles < 10°, 4–8 mm for varus angles between 10° and 20°, and 8–12 mm for varus angles > 20° [14]. The average value for each segment is about 2 mm for varus angle < 10°, 6 mm for varus angle between 10° and 20°, and 10 mm for varus angle > 20°. The results of the difference between the varus angle and the shortening height in Table 3 can be obtained by using the formula *t*' = d*sin*θ* (*d* is taken as the average value) for the difference between the femoral shortening lengths of the two osteotomy methods.

**Table 3:** Shortening height of “point-to-face” and “face-to-face” with different varus angles

Osteotomy method	Shortening length at different varus angles (mm)				
	5°	10°	15°	20°	25°
“point-to-face”	2	4	6	8	10
t'	2.11	4.21	6.27	8.28	10.25
“face-to-face”	4.11	8.21	12.27	16.28	20.25

**Fig. 10:**
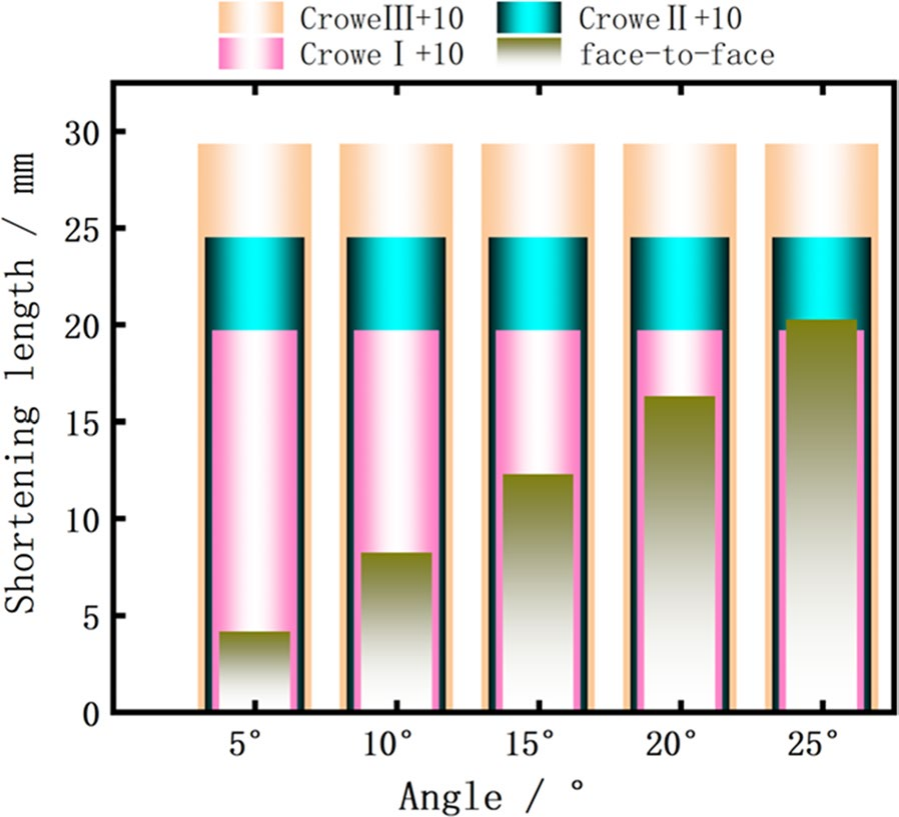
Multilayer histogram comparing the length of “face-to-face” and Crowe I, II, and III + 10 mm under different varus angles

**Fig. 11:**
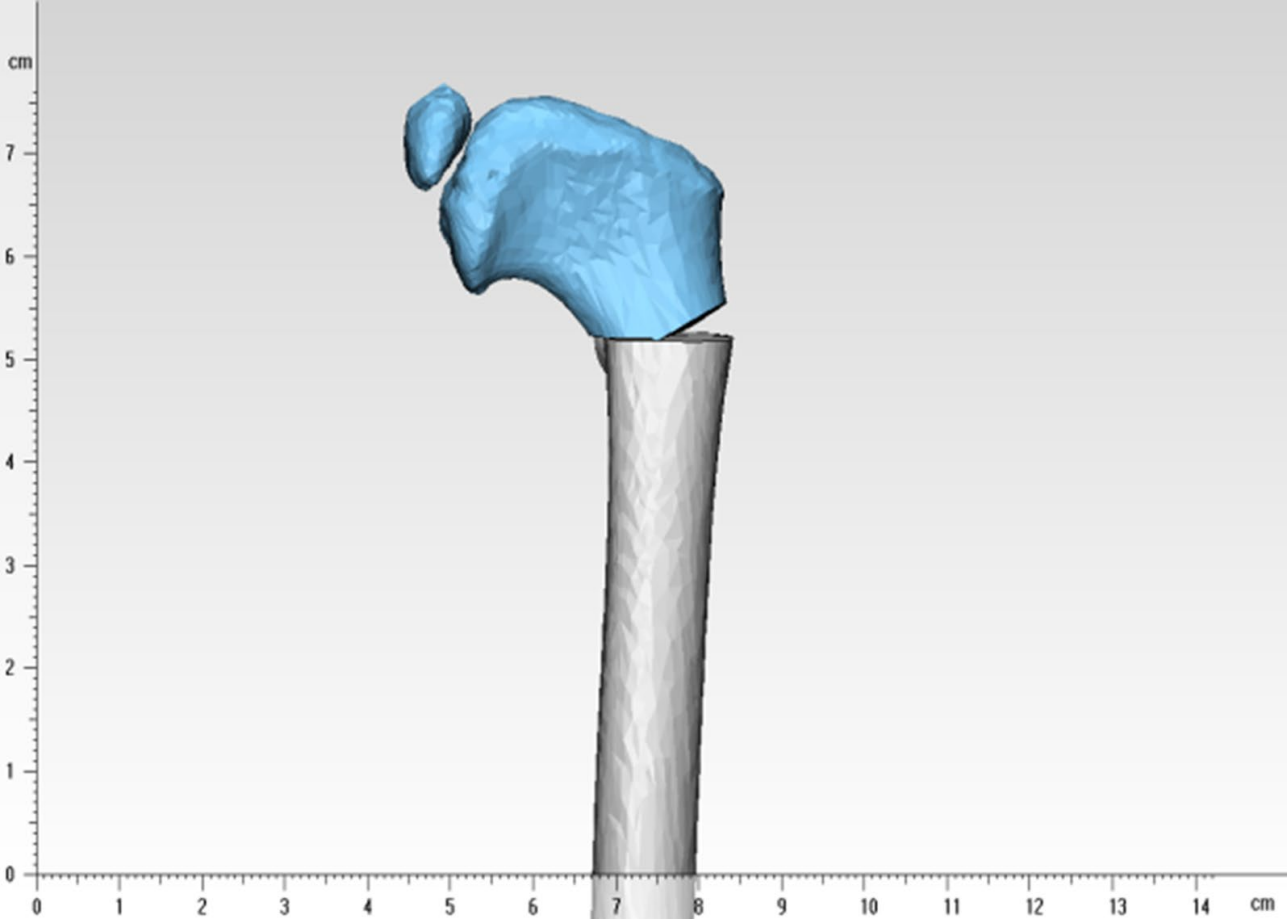
“Half-wedge” osteotomy of the proximal femur osteotomy, with a shortened length of the proximal femur between the “point-to-face” and “face-to-face” osteotomy

Using the postoperative bilateral lower limb inequality within 1 cm as a reference, we can conclude that shown in Fig. 10 and Table 4. In the “point-to-face” osteotomy, the maximum difference in the length of both lower limbs within 25° is about 10 mm; for Crowe I, II, III, and IV children, this method can be used to perform proximal femoral varus. When performing “face-to-face” osteotomy, for children with Crowe I, varus within 10° can be performed directly; when the varus angle is between 10° and 20°, we need to make a decision according to the specific dislocation height of the child; and when the varus angle is > 20°, the postoperative length difference of both lower limbs is > 10 mm, so it is not recommended. For Crowe II children, varus within 20° can be performed directly; for varus angle > 20°, we need to make a decision according to the specific dislocation height of the child. For Crowe III and IV children, the “face-to-face” varus osteotomy can be used. In cases where the “face-to-face” osteotomy is not applicable, especially in patients with unilateral DDH, we can also use a “half-wedge” osteotomy of the proximal femur (Fig. 11), which avoids excessive postoperative shortening of the lower extremity while maximizing the contact of the osteotomy surface. This avoids excessive postoperative shortening of the lower extremity while maximizing contact with the osteotomy surface and to some extent promotes bone healing at the osteotomy.

**Table 4:** Conditions for clinical application of each type

Osteotomy conditions	Crowe I	Crowe II	Crowe III	Crowe IV
“point-to-face” < 25°	✓	✓	✓	✓
“face-to-face” < 10°	✓	✓	✓	✓
“face-to-face” 10° ~ 20°	Decision making based on the specific dislocation height of the child	✓	✓	✓
“face-to-face” > 20°	×	Decision making based on the specific dislocation height of the child	✓	✓

Through the paired study of the direct measurement method and formula method and related statistical analysis, the results showed that the formula we derived is highly accurate, reproducible, and has clinical application value. At the same time, we further guided the clinical selection of the appropriate osteotomy method based on the derived results.

The shortcoming of this study is that only the difference in limb length after “point-to-point” and “face-to-face” varus osteotomy of the proximal femur was studied, and other types of osteotomy were not included; since the starting point of this study is the height of femoral dislocation, it is a direct guide for patients who are staged using the Crowe staging, which is widely used in clinical practice, while the significance of other clinical staging needs to be further investigated; proximal femoral varus osteotomy is often performed in combination with pelvic osteotomy, and the lower limb length may be indirectly extended after pelvic osteotomy due to the change in acetabular orientation. In this study, we only corrected the cervical stem angle for the proximal femur, but in clinical work, we need to correct and fix the cervical stem angle in the cervical stem plane after the correction of the anterior inclination angle, so whether the anterior femoral inclination angle has any long-term effect on the length of the patient’s limbs is also something we need to further study. This study did not consider the effect of individual differences on the trend of correction and the variation in the degree of bone growth stimulation by osteotomy. The extent to which this factor may bias our findings is the focus of our further research.

## Conclusions

The formula we derived can accurately calculate the effect of the difference proximal femoral osteotomy on leg length to aid the preoperative planning for this surgery. The results of this study are also useful for guiding clinical decision making in children with DDH.

## Data Availability

The datasets generated and/or analyzed during the current study are available from the corresponding author on reasonable request.

## Abbreviation

DDH: Developmental dysplasia of the hip
